# Pax4-Ghrelin mediates the conversion of pancreatic ε-cells to β-cells after extreme β-cell loss in zebrafish

**DOI:** 10.1242/dev.201306

**Published:** 2023-03-27

**Authors:** Junqin Yu, Jianlong Ma, Yanfeng Li, Yang Zhou, Lingfei Luo, Yun Yang

**Affiliations:** Institute of Developmental Biology and Regenerative Medicine, Southwest University, Beibei 400715 Chongqing, China

**Keywords:** β-cell regeneration, ε-cell transdifferentiation, Pax4, *Ghrelin*, Zebrafish

## Abstract

Pancreatic ε-cells producing ghrelin are one type of endocrine cell found in islets, which have been shown to influence other intra-islet cells, especially in regulating the function of β cells. However, the role of such cells during β-cell regeneration is currently unknown. Here, using a zebrafish nitroreductase (NTR)-mediated β-cell ablation model, we reveal that ghrelin-positive ε-cells in the pancreas act as contributors to neogenic β-cells after extreme β-cell loss. Further studies show that the overexpression of *ghrelin* or the expansion of ε-cells potentiates β-cell regeneration. Lineage tracing confirms that a proportion of embryonic ε-cells can transdifferentiate to β-cells, and that the deletion of Pax4 enhances this transdifferentiation of ε-cells to β-cells. Mechanistically, Pax4 binds to the *ghrelin* regulatory region and represses its transcription. Thus, deletion of Pax4 derepresses *ghrelin* expression and causes producing more ghrelin-positive cells, enhancing the transdifferentiation of ε-cells to β-cells and consequently potentiating β-cell regeneration. Our findings reveal a previously unreported role for ε-cells during zebrafish β-cell regeneration, indicating that Pax4 regulates *ghrelin* transcription and mediates the conversion of embryonic ε-cells to β-cells after extreme β-cell loss.

## Teaser

**Summary:** A previously unreported role for pancreatic ε-cells in zebrafish reveals that the conversion of ε-cells to β-cells can serve as a source of regeneration after extreme β-cell loss.

## INTRODUCTION

Ghrelin-producing ε-cells are recognised as the fifth type of endocrine cell; their locations are independent of other endocrine cells in the pancreas (Wierup et al., 2002). Human ε-cells appear in the peripheral rim of fetal islets as early as mid-gestation (Andralojc et al., 2009). Previous studies have revealed that administration of ghrelin can increases blood glucose and decreases insulin in the plasma of mammals (Dezaki et al., 2004), and a peptide derived from the C-terminal fragment of the *ghrelin* precursor can generate insulin-producing cells from mesenchymal stem cells (El-Asfar et al., 2018). Furthermore, genetic lineage analyses with a ghrelin:Cre-EGFP knock-in mouse showed that ghrelin-positive ε-cells give rise to significant numbers of α-cells and pancreatic polypeptide (PP) cells (formerly known as γ-cells) and to rare β-cells in adult islet (Arnes et al., 2012). This ghrelin-expressing lineage also contributes to subsets of exocrine and ductal cells, suggesting that some embryonic ε-cells possibly play the role of multipotent progenitors (Arnes et al., 2012). Pancreatic β-cell regeneration is a potential strategy for reversing loss of insulin production and function. Exploring multiple cell types that convert to neogenic β-cells is necessary to gain complementary insights into β-cell regeneration. Various intrapancreatic cells have been found that can contribute to neogenic β-cells, but the role of ε-cells during β-cell regeneration remains unknown.

Previously, β-cell regeneration was reported in a transgenic mice model of diphtheria toxin (DT)-induced β-cell ablation. Researchers used lineage tracing to label the glucagon-producing α-cells before β-cell ablation and found that large fractions of regenerated β-cells were derived from these α-cells (Thorel et al., 2010). Another study found that diabetes could be reversed in mouse by age-dependent conversion of pancreatic δ-cells to insulin-producing cells (Chera et al., 2014). A population of pancreatic γ-cells has also been shown to activate insulin expression in response to mouse β-cell injury (Perez-Frances et al., 2021). Furthermore, ectopic expression of Pax4 in α-cells of transgenic mice can force α-cells into converting to β-cells, suggesting that the transcription factor Pax4 specifies β-cell fate at the expense of α-cell identity (Collombat and Mansouri, 2009). Unavoidably, many studies in mouse models require constant administration of insulin to prolong survival after near-total β-cell ablation (Thorel et al., 2010; Chera et al., 2014) and the limited regeneration ability of mammals results in a lifelong reliance on insulin (Moss et al., 2009).

Nitroreductase (NTR)-mediated cell ablation in zebrafish has been reported to be an effective system for inducing the death of β-cells using the pro-drug metronidazole (MTZ) (Curado et al., 2007). A zebrafish β-cell regeneration model using the NTR/MTZ system can control β-cell ablation temporally and spatially without insulin injections to maintain zebrafish survival. In previous work, researchers performed lineage tracing for each pancreatic α-cell, β-cell and δ-cell, and showed that the ratios of α-cells contributing to newly generated β-cells are much higher than surviving β- and δ-cells (Ye et al., 2015). Evidence has revealed that Pax4 can repress pancreatic α-cell generation and knockdown of Pax4 in zebrafish embryos causes a significant increase in α-cell numbers (Djiotsa et al., 2012). Interestingly, ghrelin-expressing ε-cells, as well as α-cells, in islets are also increased in *pax4* morphants, with no apparent effect on δ-cell numbers (Djiotsa et al., 2012). Many researchers have been attempting to regenerate β cells by inducing the transdifferentiation of α-cells into β-cells (Zhang et al., 2016; Zhou and Melton, 2018), but ignore the possibility of pancreatic ε-cells (Sakata et al., 2019). By taking advantage of the short growth cycle and transparent *in vivo* visualisation of zebrafish embryos, we have used a NTR/MTZ-induced β-cell ablation model to explore the role of ε-cells in β-cell regeneration.

## RESULTS AND DISCUSSION

### A fraction of ghrelin-positive ε-cells expresses insulin after extreme β-cell loss

**Fig. 1.**
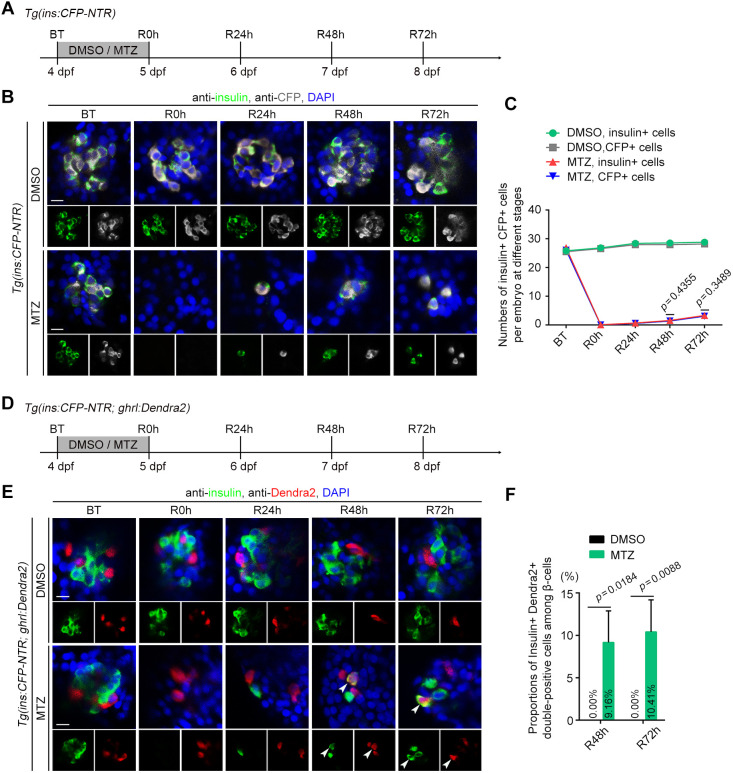
**A small proportion of ghrelin-positive ε-cells express insulin after near-total β-cell ablation.** (A) Experimental scheme for DMSO or MTZ treatment of the *Tg(ins:CFP-NTR)* larvae. (B) Antibody staining of insulin and CFP showed that pancreatic β-cells in zebrafish regenerated slowly after near-total ablation. (C) The numbers of insulin^+^ and CFP^+^ cells per embryo at different stages (*n*=22). (D) Experimental scheme for DMSO or MTZ treatment of the *Tg(ins:CFP-NTR; ghrl:Dendra2)* larvae. (E) A small proportion of ε-cells expressed insulin during β-cell regeneration. Arrowheads indicate CFP^+^ Dendra2^+^ double-positive cells. (F) Statistical diagram of proportions of insulin^+^ Dendra2^+^ double-positive cells among β-cells (*n*=20, the average percentage is shown in the histogram). All statistical data are expressed as mean±s.e.m. *P*-values were calculated using an unpaired Student's *t*-test. Scale bars: 10 µm. BT, before treatment; R0 h, R24 h, R48 h and R72 h indicate regeneration for 0, 24, 48 and 72 h, respectively.

To investigate the zebrafish β-cell regeneration process, the *Tg(ins:CFP-NTR)* transgenic line (Wang et al., 2020) was used, in which the cyan fluorescent protein (CFP) is fused to NTR and driven by β-cell-specific promoter *insulin* (*ins*). A combination of CFP labelling and insulin antibodies was used to detect the efficiency and specificity of β-cell ablation. Treating *Tg(ins:CFP-NTR)* larvae with MTZ at 4-5 days post-fertilisation (dpf) for 24 h near-totally ablated insulin^+^ and CFP^+^ cells (Fig. 1A-C). We the washed out MTZ and collected the larvae from before treatment (BT) to regeneration for 72 h (R72 h). The results showed that a few neogenic insulin^+^ CFP^+^ double-positive cells were present in the MTZ-treated group (Fig. 1B,C), indicating that zebrafish pancreatic β-cells could regenerate slowly after near-total ablation.

To analyse the role of ghrelin-positive ε-cells during β-cell regeneration in zebrafish, the *Tg(ghrl:Dendra2)^cq151^* transgenic line was generated, using a photoconvertible green fluorescent protein Dendra2 driven by a promoter of *ghrelin* (*ghrl*). In addition, to verify the specificity of this promoter, we generated an endogenous *ghrelin* gene integrity-maintaining and intron-targeting knock-in zebrafish line, *Ki(ghrl-p2a-Tomato)*, by CRISPR/Cas9 (Fig. S1A-C). Living images and statistics showed that almost every Tomato-labelled *ghrelin*-expressing ε-cell in *Ki(ghrl-p2a-Tomato)* was colocalised with a ghrl:Dendra2-labelled cell at 30 hpf (Fig. S1D,E). After that, by treating the larvae in the *Tg(ins:CFP-NTR; ghrl:Dendra2)* double transgenic background with MTZ (Fig. 1D), we found the Dendra2-labelled ε-cells independently presented in islets and did not overlap with insulin-labelled cells in the DMSO control group. However, in the MTZ-treated group, a small proportion of ε-cells could express insulin during β-cell regeneration (Fig. 1E,F), which implies that ghrelin-positive ε-cells possibly contribute to neogenic β-cells after extreme β-cell loss.

### Overexpression of *ghrelin* or expansion of ε-cells potentiates β-cell regeneration

**Fig. 2.**
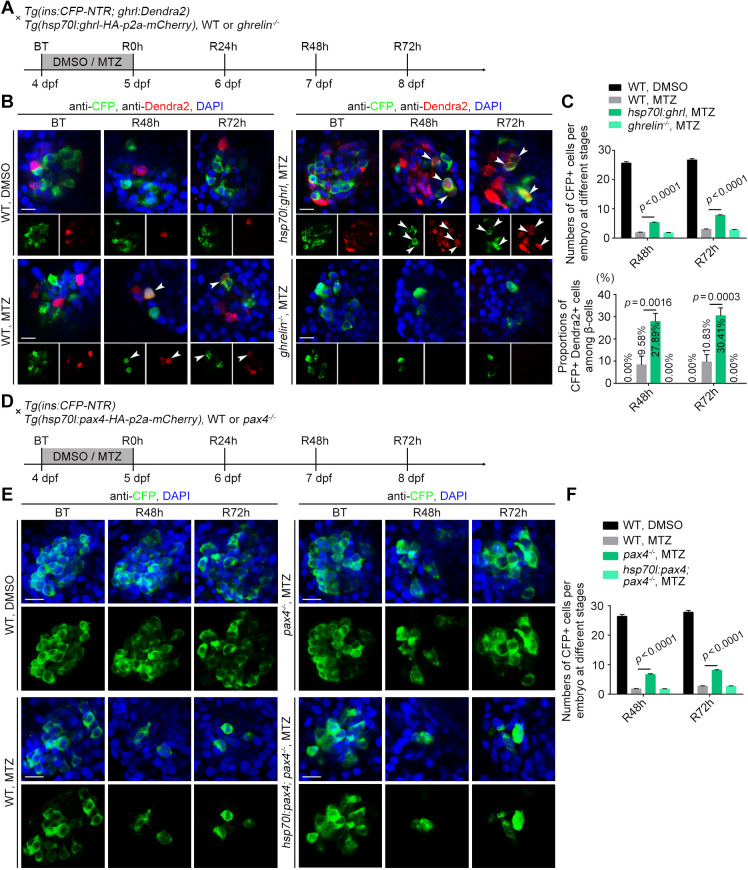
**Overexpression of *ghrelin* or deletion of Pax4 potentiates β-cell regeneration.** (A) Experimental scheme for DMSO or MTZ treatment of the wild-type, *ghrelin^−/−^* or *Tg(hsp70l:ghrl-HA-p2a-mCherry)* larvae under the *Tg(ins:CFP-NTR; ghrl:Dendra2)* background. (B) Overexpression of *ghrelin* led to an increased number of neogenic CFP^+^ cells, including Dendra2^+^ CFP^+^ double-positive cells (arrowhead) compared with the wild-type MTZ-treated group, but not in the *ghrelin^−/−^* mutant. (C) Numbers of CFP^+^ cells and proportions of Dendra2^+^ CFP^+^ double-positive cells per embryo at different stages (*n*=20). (D) Experimental scheme for DMSO or MTZ treatment of the wild-type, *pax4^−/−^* or *Tg(hsp70l:pax4-HA-p2a-mCherry)* larvae under the *Tg(ins:CFP-NTR)* background. (E) The numbers of neogenic CFP^+^ cells were significantly increased in the *pax4^−/−^* mutant, and overexpression of *pax4* could rescue the increased β-cell regeneration in the *pax4^−/−^* mutant. (F) The numbers of CFP^+^ cells per embryo at different stages (*n*=20). All statistical data are expressed as mean±s.e.m. *P*-values were calculated using an unpaired Student's *t*-test. Scale bars: 10 µm.

We crossed the *Tg(ins:CFP-NTR; ghrl:Dendra2)* transgenic line with the heat shock-inducible line *Tg(hsp70l: ghrl-HA-p2a-mCherry)^cq152^* for conditional *ghrelin* overexpression (Fig. S2A) and treated their offspring with MTZ and heat-shocked every day from 3 dpf (Fig. 2A and Fig. S2A). Heat shock-inducible overexpression of *ghrelin* caused an increased number of Dendra2^+^ cells expressing *ghrelin* in islets. In addition, levels of neogenic CFP^+^ β-cells, including ghrl:Dendra2^+^ and ins:CFP^+^ double-positive cells, were also increased when compared with the wild-type MTZ-treated group from R48 h and R72 h (Fig. 2B,C). These data suggest that overexpression of *ghrelin* could potentiate β-cell regeneration after extreme β-cell loss. Moreover, we crossed the *Tg(ins:CFP-NTR; ghrl:Dendra2)* transgenic line with a *ghrelin^−/−**,**cq153^* mutant generated using CRISPR/Cas9 (Fig. S2B-D) and treated the offspring with MTZ from 4 to 5 dpf (Fig. 2A). The results showed Dendra2^+^ cells expressing *ghrelin* were totally lost in the *ghrelin^−/−^* mutant, but the number of neogenic CFP^+^ β-cells was not a significant changed compared with the wild-type MTZ-treated group (Fig. 2B,C). We reasoned that other intra-islet cells should also contribute to neogenic β-cells during β-cell regeneration. To investigate this, we used the *Tg(gcga:DsRed)^cq160^*, *Tg(sst2:DsRed)^cq61^* transgenic line or *sst1.1* antisense probes to detect the contribution of α-cell and two types of δ-cell that have been reported to be heterogeneous in zebrafish (Singh et al., 2022). Analysis of regeneration showed that some gcga^+^ α-cells and sst1.1^+^ δ-cells, but not sst2^+^ δ-cells, could express ins:CFP during β-cell regeneration (Fig. S3A and S3B). However, these cells were not colocalised with ghrl:Dendra2^+^ and ins:CFP^+^ double-positive cells (Fig. S3A,B), implying that ghrelin-positive ε-cells might have an independent role in β-cell regeneration in larval zebrafish.

Because expression of *ghrelin* is upregulated in zebrafish *pax4* morphants (Djiotsa et al., 2012) and expression of ghrelin-positive cells is expanded in mouse *Pax4* mutants (Wang et al., 2008), we used CRISPR/Cas9 to generate a *pax4^−/−**,**cq154^* zebrafish mutant (Fig. S4A-C) to confirm whether Pax4 affects the role of ε-cells during β-cell regeneration. By treating *pax4^−/−^* mutant under *Tg(ins:CFP-NTR)* genetic background with MTZ (Fig. 2D), we found that the number of neogenic CFP^+^ β-cells was significantly increased compared with the wild-type MTZ-treated group at R48 h and R72 h (Fig. 2E,F). Furthermore, we generated a heat-shock-inducible line *Tg(hsp70l:pax4-HA-p2a-mCherry)^cq155^* for conditional *pax4* overexpression (Fig. S4D). Treatment of the *pax4^−/−^* mutant in the *Tg(ins:CFP-NTR; hsp70l:pax4-HA-p2a-mCherry)* genetic background with MTZ and a heat-shock each day from 3 dpf (Fig. 2D and Fig. S4D) revealed that overexpression of *pax4* could rescue the increased β-cells regeneration in *pax4^−/−^* mutant (Fig. 2E,F). These data show that knockout of *pax4* could potentiate β-cells regeneration after extreme β-cell loss, suggesting this incremental β-cell regeneration might be associated with the expansion of ghrelin-positive cells in *pax4^−/−^* mutant.

### Deletion of Pax4 enhances the transdifferentiation of ε-cells to β-cells

**Fig. 3.**
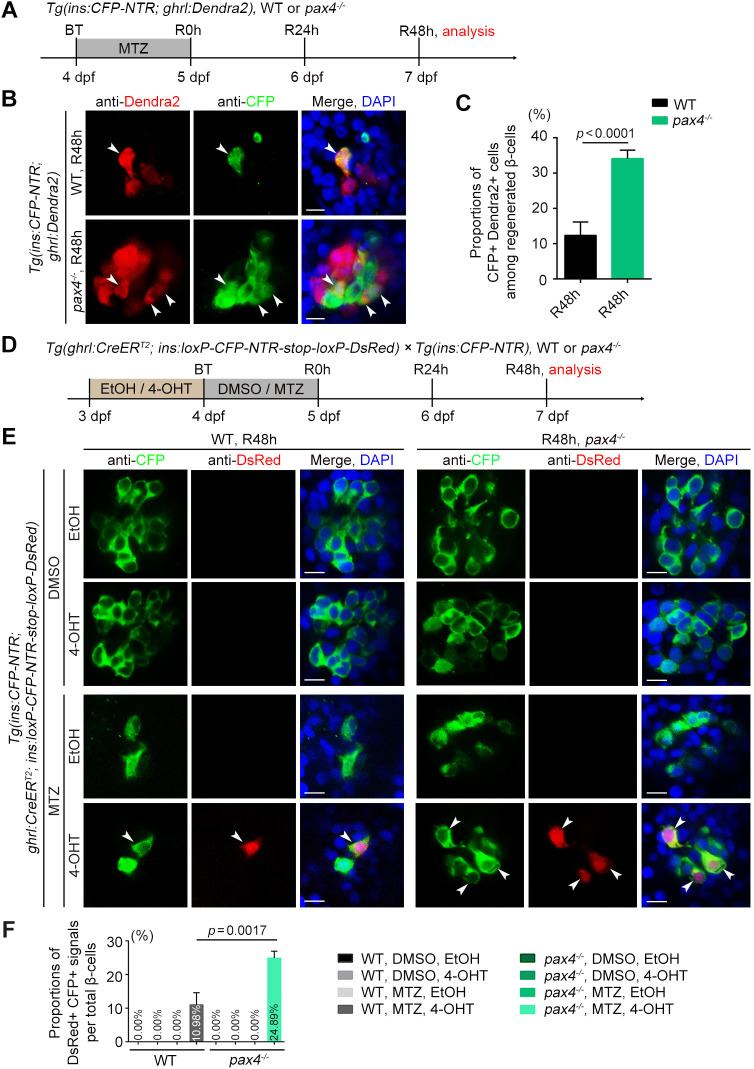
**Deletion of Pax4 causes the increased proportion of ε-cells converting to β-cells during β-cell regeneration.** (A) Experimental scheme for MTZ treatment of the wild-type or *pax4^−/−^* mutant under the *Tg(ins:CFP-NTR; ghrl:Dendra2)* double transgenic background. (B) More neogenic CFP^+^ β-cells overlapped with the Dendra2^+^ ε-cells in *pax4^−/−^* mutant relative to the wild-type MTZ-treated group. Arrowheads indicate CFP^+^ Dendra2^+^ double-positive cells. (C) Statistical diagram of proportions of CFP^+^ Dendra2^+^ double-positive cells among regenerated β-cells (*n*=25). (D) Experimental scheme for DMSO, MTZ, ethanol or 4-OHT treatment of the wild-type or *pax4^−/−^* mutant under *Tg(ghrl:CreER^T2^; ins:loxP-CFP-NTR-stop-loxP-DsRed; ins:CFP-NTR)* triple transgenic background. (E) Antibody staining of CFP and DsRed after 48 h of regeneration (R48 h) showed an increased proportion of DsRed-labelled cells (arrowheads) in the *pax4^−/−^* MTZ- and 4-OHT-treated group compared with the wild-type group. (F) The proportions of DsRed^+^ CFP^+^ signals per total β-cells (*n*=22). All statistical data are expressed as mean±s.e.m. *P*-values were calculated using an unpaired Student's *t*-test. Scale bars: 10 µm.

In view of the above data, we speculated that ghrelin-positive ε-cells might transdifferentiate to β-cells after extreme β-cell loss. To test this hypothesis, at the outset, we treated the *pax4^−/−^* mutants under the *Tg(ins:CFP-NTR; ghrl:Dendra2)* double transgenic background with MTZ (Fig. 3A). Analysis of regeneration showed that more CFP^+^ β-cells overlapped with the Dendra2^+^ ε-cells in *pax4^−/−^* mutant, and that the proportion of these double-positive cells among neogenic cells was statistically higher than in the wild-type MTZ-treated group at R48 h (Fig. 3B,C). Slc2a2 (also known as Glut2) is the major glucose transporter that mediates glucose uptake into β-cells, leading to insulin secretion (Michau et al., 2013). This functional marker of β-cells could be detected in CFP^+^ Dendra2^+^ double-positive cells of the wild-type MTZ-treated group, and its expression was also upregulated in response to the increase in neogenic β-cells in the *pax4^−/−^* mutant (Fig. S5A-C). Pdx1 and Sox9 are pancreatic progenitor markers involved in early pancreatic differentiation (Ma et al., 2022; Seymour et al., 2012; Kimmel et al., 2011). Under the same treatment as before (Fig. S5A), we found that these two transcription factors were expressed in insulin/CFP^+^ and ghrl:Dendra2^+^ double-positive cells and exhibited higher expression levels in *pax4^−/−^* mutants (Fig. S5D-G), implying that these double-positive cells might be a bipotential intermediate during β-cell regeneration. Thus, ghrelin-positive ε-cells possibly contribute to neogenic β-cells through ε-cell to β-cell transdifferentiation after extreme β-cell loss.

To confirm whether the neogenic β-cells arise from transdifferentiation of ε-cells, we generated a *Tg(ghrl:CreER^T2^)^cq156^* transgenic line and used the 4-hydroxytamoxifen (4-OHT)-dependent Cre/loxP system to perform conditional lineage analysis. First, this transgenic line was crossed with *Tg(ghrl:Dendra2)* and *Tg(β-actin:loxP-stop-loxP-DsRed)^cq159^* or *Tg(β-actin:loxP-DsRed-stop-loxP-GFP)^cq39^*, and their offspring were treated with 4-OHT to detect the efficiency and specificity of ghrl:CreER^T2^. These data showed that the majority of the ghrl:CreER^T2^-dependent fluorescent signals overlapped with ghrl:Dendra2^+^ ε-cells (Fig. S6A-C), and that these signals did not label insulin^+^ β-cells (Fig. S6B,C) and other intrapancreatic cells (Fig. S6D,E). Next, we crossed the *Tg(ghrl:CreER^T2^)* transgenic line with *Tg(ins:loxP-CFP-NTR-stop-loxP-DsRed)^cq67^* and *Tg(ins:CFP-NTR)* and treated their offspring with 4-OHT or ethyl alcohol (EtOH) from 3 to 4 dpf then with MTZ or DMSO from 4 to 5 dpf (Fig. 3D). In contrast to the wild-type DMSO-treated and MTZ-control group at R48 h, a small proportion of neogenic CFP^+^ cells overlapped with DsRed^+^ cells in the wild-type MTZ- and 4-OHT-treated group (Fig. 3E,F), indicating that some wild-type ε-cells had a degree of plasticity and could convert to β-cells. When the number of ε-cells was expanded in the *pax4^−/−^* mutant, the proportion of DsRed^+^ CFP^+^ double-positive cells was statistically increased in the *pax4^−/−^* MTZ- and 4-OHT-treated groups (Fig. 3E,F). These data indicate that deletion of Pax4 can enhance the transdifferentiation of ε-cells into β-cells after extreme β-cell loss, suggesting that Pax4 acts as an upstream mediator in converting ε-cells to β-cells.

### Pax4 binds to the *ghrelin* regulatory region and represses its transcription

**Fig. 4.**
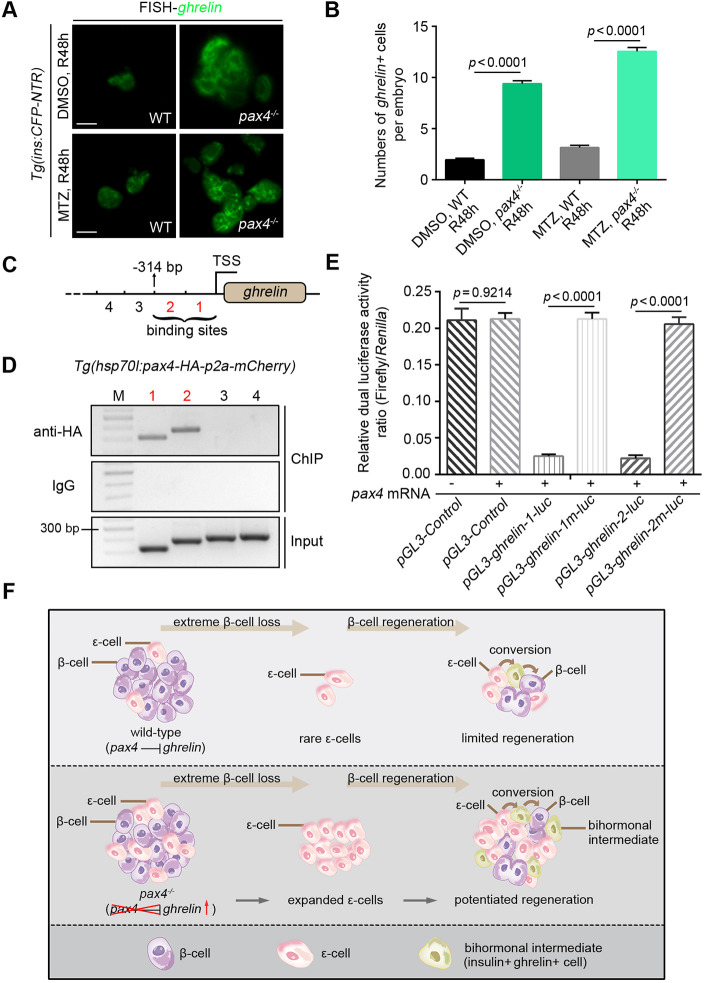
**Pax4 binds to genomic DNA at the *ghrelin* regulatory locus to repress its transcription.** (A) Under the *Tg(ins:CFP-NTR)* transgenic background, fluorescent *in situ* hybridisation of the *ghrelin* probe showed that the *pax4^−/−^* mutant exhibited a significant expansion of ghrelin-expressing ε-cells both in the DMSO-control and MTZ-treated group compared with the wild-type after 48 h of regeneration (R48 h). (B) Statistical diagram of numbers of *ghrelin*^+^ cells per embryo at R48 (*n*=20). (C,D) Two consecutive sequence fragments 314 bp upstream of the *ghrelin* TSS were identified as the binding sites of Pax4 (C, fragments 1 and 2), which were shown by ChIP analyses (D, bands 1 and 2). Serial numbers represent the corresponding DNA fragments upstream of the *ghrelin* TSS. (E) Activities of the luciferase reporter driven by the *ghrelin-1* and *ghrelin-2* sequences, but not the corresponding mutated sequences (*ghrelin-1m* and *ghrelin-2m*), were reduced by *pax4* mRNA (*n*=6). (F) Schematic illustration of the conversion of pancreatic ε-cells to β-cells mediated by Pax4-ghrelin after extreme β-cell loss in zebrafish. All statistical data are expressed as mean±s.e.m. *P*-values were calculated using an unpaired Student's *t*-test. Scale bars: 10 µm.

Zebrafish *pax4^−/−^* mutants exhibited a significant expansion of ghrelin-positive ε-cells both in the DMSO-control and MTZ-treated group (Fig. 4A,B). As Pax4 is a transcription factor, we investigated whether it controls the expression of *ghrelin*. Two consecutive sequence fragments in the regulatory region of *ghrelin*, 314 bp upstream of its transcriptional starting site (TSS) (hereafter called *ghrelin*-1 and *ghrelin*-2) were identified as potential binding sites of Pax4 (Fig. 4C). Chromatin immunoprecipitation (ChIP) from *Tg(hsp70l:pax4-HA-p2a-mCherry)* embryos an the association of Pax4-HA with genomic DNA at the *ghrelin-*1 and *ghrelin*-2 loci (Fig. 4D). Activities of the luciferase reporters driven by the *ghrelin-*1 and *ghrelin*-2 sequences, but not by the corresponding mutated sequences, were reduced by overexpression of *pax4* (Fig. 4E). These results show that, in zebrafish embryos, Pax4 binds to genomic DNA at the regulatory region of *ghrelin* gene to repress its expression, restricting the conversion of ε-cells to β-cells. In summary, our findings reveal that, in the wild type, the number of ghrelin-positive ε-cells is small, but these cells are still capable of transdifferentiation to β-cells after near-total β-cell ablation. Deletion of Pax4 can derepress *ghrelin* expression to expand the number of ghrelin-positive ε-cells in the intra-islet, enhancing the transdifferentiation of ε-cells to β-cells and consequently potentiating β-cells regeneration (Fig. 4F).

In previous reports, the *ghrelin* gene-derived peptide has ben shown to enhance *in vitro* generation of islets (Baragli et al., 2013). Ghrelin released by ε-cells prevents the induction of diabetes in streptozotocin-treated rats (Irako et al., 2006; Granata et al., 2010, 2012). Here, we have found that, after near-total β-cell ablation, overexpression of *ghrelin* can potentiate β-cell regeneration (Fig. 2B,C). Deletion of Pax4 caused the derepression of *ghrelin* (Fig. 4E), which also could potentiate β-cell regeneration (Fig. 2E,F). These enhancements of β-cell regeneration were based on increased ghrelin-expressing cells in islets (Figs 2B and 4A). However, loss of ghrelin did not affect β-cell regeneration (Fig. 2B,C), which implies that sufficient ghrelin and the presence of ε-cells in islets are a prerequisite for the transdifferentiation of ε-cells to β-cells.

Pancreatic ε-cells are vital sources of ghrelin during human fetal development, but their numbers decrease in adults and subsequently increase in the stomach (Andralojc et al., 2009; Wierup et al., 2002). In mice, ghrelin-producing ε-cells appear at embryonic days 8.5-10.5 (E8.5-10.5), which is equivalent to gestational weeks 8-9 in humans. This is earlier than other islet cell types (Heller et al., 2005; Prado et al., 2004). The mechanism by why pancreatic ε-cells increase during the embryonic stage and decrease before birth is unclear. It has previously been reported that Pdx1 and Ngn3 are essential factors in pancreatic endocrine specification, and that their expression peaks at the same time as that of ε-cell generation (Apelqvist et al., 1999; Sakata et al., 2019; Zhu et al., 2017). These observations imply that, during endocrine development, ε-cells are possibly intermediate cells with potential and not terminally differentiated cells. The role of ε-cells as multipotent progenitors that convert to other intrapancreatic cells in mouse embryos supports this (Arnes et al., 2012).

Zebrafish ghrelin is already expressed at 6 hpf, before hatching and feeding (Eom et al., 2013), which suggests that it might have a developmental role. Moreover, we found that the ghrelin-producing ε-cells in pancreas were most abundant at 24-30 hpf (Fig. S1D,E) and slowly reduced from 3 to 4 dpf (Fig. 1E,F), which is similar to the progress of mammalian ε-cells, which initially increase then decrease in pancreas. In this study, Cre/loxP-based inducible lineage tracing showed that a fraction of ε-cells has the ability to transdifferentiate to β-cells after extreme β-cell loss (Fig. 3E,F). Albeit in a low proportion, they may represent a small population of ε-cells with expanded potential during the early developmental stages in zebrafish. This potential plasticity of embryonic ε-cells might support β-cell regeneration but does not exclude other cell types that convert to β-cells after β-cell injury (Chera et al., 2014; Perez-Frances et al., 2021; Carril Pardo et al., 2022), such as gcga^+^ α-cells and sst1.1^+^ δ-cells (Fig. S3B).

Deletion of Pax4 expands the numbers of ε-cells (Fig. 4A,B) and enhances the transdifferentiation of ε-cells to β-cells (Fig. 3D-F). During early embryonic development in zebrafish, knockdown of Pax4 causes a significant increase in α-cell numbers (Djiotsa et al., 2012); it is unknown whether this enhances zebrafish α-cell to β-cell conversion. However, the contribution of α-cells and δ-cells to some of neogenic β-cells did not influence the role of ε-cells during β-cell regeneration (Fig. S3A,B). Besides, zebrafish pancreatic expression of *ghrelin* is slightly increased in *pax4* morphants (Djiotsa et al., 2012), but it is more significant in *pax4^−/−^* mutants (Fig. 4A,B).

Compared with adult β-cell regeneration models, most work exploring intra-islet cell transdifferentiation and endogenous β-cell neogenesis during embryonic and larval development still relies on the potential plasticity of different pancreatic cell types. However, it is gradually being accepted and, hopefully, translated to humans, with the discovery of specific cellular heterogeneity and potentially intermediate stages in single-cell RNAseq data from human islets (Baron et al., 2016; Segerstolpe et al., 2016). Moreover, the acquisition of zebrafish β-cell functionality begins as early as islet vascularisation at 72 hpf (Zhao et al., 2019), maturing earlier than in mice. Here, through the NTR/MTZ zebrafish model, our findings reveal that the conversion of ε-cells to β-cells can replenish β-cells after near-total β-cell ablation, providing a new perspective for β-cell regeneration using strategies that target ε-cells.

## MATERIALS AND METHODS

### Zebrafish strains

The zebrafish facility and study were approved by the Institutional Review Board of Southwest University (Chongqing, China). Zebrafish were maintained under standard laboratory conditions according to IACUC protocols. Embryos were treated with 0.003% 1-phenyl-2-thiourea (PTU, Sigma) to inhibit pigment formation.

The *Tg(ins:CFP-NTR)* (Wang et al., 2020), *Tg(ghrl:Dendra2)^cq151^*, *Tg(hsp70l:ghrl-HA-p2a-mCherry)^cq152^*, *Tg(gcga:DsRed)^cq160^*, *Tg(sst2:DsRed)^cq61^*, *Tg(hsp70l:pax4-HA-p2a-mCherry*)*^cq155^*, *Tg(ghrl:CreER^T2^*; *cryaa:Cerulean)^cq156^*, *Tg(β-actin:loxP-stop-loxP-DsRed)^cq159^*, *Tg(β-actin:loxP-DsRed-stop-loxP-GFP)^cq39^* (Chen et al., 2019) and *Tg(ins:loxP-CFP-NTR-stop-loxP-DsRed)^cq67^* transgenic lines, *Ki(ghrl-p2a-Tomato)* knock-in line and *ghrelin^−/−,cq153^*, *pax4^−/−,cq154^* mutants were established or used in this study.

### Generation of transgenic lines

To generate the pBluescript-*ghrl:Dendra2*, pBluescript-*ghrl:CreER^T2^*, pBluescript-*hsp70l:ghrl-HA-p2a-mCherry*, pBluescript-*gcga:DsRed*, pBluescript-*sst2:DsRed* and pBluescript-*hsp70l:pax4-HA-p2a-mCherry* plasmids, the *Dendra2* and *CreER^T2^* fragments were subcloned downstream of the *ghrl* (−3.6 k) promoter, the *DsRed* fragment was subcloned downstream of the *gcga* (−2.7 k) or *sst2* (−2.5 k) promoter, and the *ghrelin-HA-p2a-mCherry* and *pax4-HA-p2a-mCherry* fragments were subcloned downstream of the hsp70 l promoter. Constructs flanked by the *I*-SecI restriction sites were co-injected with *I*-SecI (NEB) enzyme into zebrafish embryos of the AB genetic background at the one-cell stage for transgenesis as previously described (He et al., 2014; Chen et al., 2021). Primers used for cloning: *ghrl* (−3.6 k) promoter (forward, 5′-CAATCAGTTAACGTTAAACAGTG-3′; reverse, 5′-CCTGGGAAATCTGGTATCGTTC-3′), *gcga* (−2.7 K) promoter (forward, 5′-TTGCGTTAAAAATCTACTCTGAC-3′; reverse, 5′- TTTAACAGCTGAGTCTTCCAACAC-3′), *sst2* (−2.5 K) promoter (forward, 5′-CCTCTATGTCCTTCGTCTTATTG-3′; reverse: 5′-TTCTGCTGCTTCTTTAACTCAGAAC-3′), *ghrelin* full-length CDS (forward, 5′-ATGCCTCTGAGGTGCCGTGCC-3′; reverse, 5′-GAACTCGAAAGAAGAGTCTCTAAG-3′) and *pax4* full-length CDS (forward, 5′-ATGCGCAAACCTCCCAGCAATG-3′; reverse, 5′-AAACATTGGACTATTTTGTCCAG-3′).

### Generation of the knock-in zebrafish line

Intron targeting-mediated knock-in in zebrafish was carried out as previously described (Li et al., 2015). The CRISPR/Cas9 target sequence of *ghrelin-intron* gRNA (5′-GTCACCTACTAATTCAGTGCCGG-3′) (PAM site underlined) was located in intron 3 of *ghrelin* genomic DNA. The *ghrl-p2a-Tomato* fragment consisting of the upstream of the 5′ side of the gRNA target site in intron 3, the whole last exon 4 and a *p2a-Tomato*-coding sequence was ligated into the pGEM-T vector as donor plasmid. Cas9 mRNA (500 pg), *ghrelin-intron* gRNA (100 pg) and donor plasmid (30 pg) were co-injected into one-cell stage fertilised zebrafish eggs. Embryos co-injected with effective gRNA were raised to adult (F0). F0 zebrafish were screened by out-crossing with AB lines. To identify the founder, the genome pool of lysed embryos was used as the template to amplify the 5′ and 3′ junction fragments of targeted genes with primers F1 (5′-GGTGGCATTAACAGCATTCTCAGCA-3′), R1 (5′-TTACTTGTACAGCTCGTCCATGCCG-3′), F2 (5′-GCCCCAGATTACAAATGCATTCTGG-3′) and R2 (5′-CTCTGTATCATCACATGAAAGTTTG-3′). Offspring of *Ki(ghrl-p2a-Tomato)* founder candidates were screened by Tomato signal at 30 hpf.

### Generation of zebrafish CRISPR mutants

The CRISPR/Cas9 target sequences of *ghrelin* gRNA (5′-GCTCACAGACTCGAGACACA AGG-3′) and *pax4* gRNA (5′-GATGATTGAGCTGGCGACTGAGG-3′) (PAM site underlined) were located at exon 1 and exon 2, respectively. Cas9 mRNA, *pax4* gRNA and *ghrelin* gRNA were separately synthesised as previously described (He et al., 2019; Cai et al., 2021). Cas9 mRNA (300 pg) and *ghrelin* gRNA/*pax4* gRNA (100 pg) were co-injected into zebrafish embryos of the AB genetic background at the one-cell stage, and then the lysates of ∼10 embryos at 24 hpf were used as the template for PCR with primers *ghrelin* (forward, 5′-TGAAAGGCAGATGCTGGTGTC-3′; reverse, 5′-TCTTTGATCACTGGTATCTCTGG-3′), *pax4* (forward, 5′-AGTGATGCCAAACTTATAATCCGT-3′; reverse, 5′-TAACACAGCCGTTGGAGACC-3′). PCR products were sequenced to detect the indels at *pax4* or *ghrelin* target region. Embryos co-injected with effective *pax4* gRNA or *ghrelin* gRNA were raised to adult (F0). F0 zebrafish were screened to identify the founder whose progeny carried the indels in the *pax4* or *ghrelin* gene. The identified founders were crossed back to the AB genetic background for three generations to obtain *pax4* or *ghrelin* stable mutant lines.

### Genotyping the *ghrelin^−/−^* mutant and *pax4^−/−^* mutant

For *ghrelin^−/−^* mutant genotyping, a 422 bp genomic DNA fragment was amplified with the primers *ghrelin* (forward, 5′-TGAAAGGCAGATGCTGGTGTC-3′; reverse, 5′-TCTTTGATCACTGGTATCTCTGG-3′), and then sequenced. A 12 bp deletion (5′-TCTTTCCTTGTG-3′) and a 5 bp (5′-CTCTT-3′) insertion were identified in the *ghrelin^−/−^* mutant. For *pax4^−/−^* mutant genotyping, a 645 bp genomic DNA fragment was amplified with the primers *pax4* (forward, 5′-AGTGATGCCAAACTTATAATCCGT-3′; reverse, 5′-TAACACAGCCGTTGGAGACC-3′), and then sequenced. A 1 bp deletion (5′-A-3′) and an 18 bp insertion (5′-GGATTGAGACCATGTGAG-3′) were identified in the *pax4^−/−^* mutant.

### Heat-shock treatment

From 3 dpf to 8 dpf, the *Tg(hsp70l:ghrl-HA-p2a-mCherry)* or *Tg(hsp70l:pax4-HA-p2a-mCherry)* transgenic embryos were heat-shocked at 38.5°C for 30 min per day and then incubated at 28.5°C until the time-point of analyses.

### MTZ treatment

The *Tg(ins:CFP-NTR)* and *Tg(ins:loxP-CFP-NTR-stop-loxP-DsRed)* transgenic larvae at 4-5 dpf were incubated with 15 mM metronidazole (MTZ, Sangon Biotech) in egg water mixed with 0.2% dimethyl sulfoxide (DMSO). Larvae were washed three times using fresh egg water after MTZ treatment for 24 h, and then incubated in egg water with PTU until the time-point of analyses. The larvae incubated in egg water with 0.2% DMSO were used as a control.

### 4-Hydroxytamoxifen treatment

For conditional induction of CreER^T2^ activity, the transgenic lines *Tg(ghrl:CreER^T2^)* were crossed to *Tg(β-actin:loxP-stop-loxP-DsRed)*, *Tg(β-actin:loxP-DsRed-stop-loxP-GFP)* or *Tg(ins:loxP-CFP-NTR-loxP-stop-DsRed)* and *Tg(ins:CFP-NTR)*. 4-Hydroxytamoxifen (4-OHT) (Sigma) was dissolved in 100% ethanol to prepare a stock concentration of 10 mM. The offspring were treated with 5 µM 4-OHT in egg water at the indicated time, washed three times using fresh egg water, and then incubated in egg water with PTU until the time-point of analyses. Relatively diluted ethanol was used as vehicle control.

### Confocal imaging

The living or fixed embryos were embedded in 35 mm glass bottom dishes using 1% low melting point agarose as previously described (Liu et al., 2016; Yang et al., 2021). Images were captured using a 20× water immersion objective mounted on the LSM780 confocal microscope. Three-dimensional images were generated by *z*-stacks using ZEN2010 software (Carl Zeiss).

### Antibody staining and *in situ* hybridisation

Zebrafish embryos were fixed overnight in 4% formaldehyde solution. Fluorescent *in situ* hybridisation (FISH), and combination of FISH and antibody staining were performed as previously described (He et al., 2020; Yang et al., 2022) using *sst1.1* (Kinkel et al., 2008) *slc2a2* (Marín-Juez et al., 2015), *pdx1*(Gao et al., 2019) and *ghrelin* antisense probes labelled with digoxigenin; anti-digoxigenin-POD (1:1000, Roche, 11633716001), anti-Dendra2 (1:1000, Evrogen, AB821), anti-CFP (1:1000, Abcam, ab6658) and anti-insulin (1:200, Abcam, ab210560) antibody; and tyramide signal amplification and fluorescence detection system (TSA, Perkin Elmer). Primers used for probe synthesis were: *ghrelin* (forward, 5′-GATGCCTCTGAGGTGCCGTG-3′; reverse, 5′-GTAATACGACTCACTATAGTCAGAACTCGAAAGAAGAGTC-3′; the T7 promoter sequence is underlined).

Zebrafish whole-mount antibody staining was carried out as previously described (Yang et al., 2021) using anti-Dendra2 (1:1000, Evrogen, AB821), anti-insulin (1:200, Abcam, ab210560), anti-CFP (1:1000, Abcam, ab6658), anti-DsRed (1:300, Santa Cruz Biotechnology, sc-101526) and anti-Sox9 (1:300, Bimake, A5080) primary antibodies, and Alexa 488-conjugated donkey anti-rabbit IgG (1:1000, Invitrogen, A-21206), Alexa 568-conjugated donkey anti-mouse IgG (1:1000, Invitrogen, A10037) and Alexa 647-conjugated donkey anti-goat IgG (1:1000, Invitrogen, A32849) secondary antibodies. The nuclei were stained with DNA fluorochrome 4′,6-diamidino-2-phenylindole (DAPI, Sigma).

### Chromatin immunoprecipitation

The *Tg(hsp70l:pax4-HA-p2a-mCherry)* embryos were heat-shocked at 26 hpf, then collected and dissociated in the lysis buffer. The chromatin was sheared by sonication to an average fragment size of 150-300 bp. Immunoprecipitations were performed as previously described (Zhang et al., 2022) using 4 µg of anti-HA (Abcam, ab9110) antibodies. IgG (Abcam) immunoprecipitation as a control sample. These chromatin immunoprecipitation (ChIP) samples and corresponding inputs were detected by PCR using primers: *ghrelin-1* (forward, 5′-GGCAGATGCTGGTGTCAGAC-3′; reverse, 5′-CCTGGGAAATCTGGTATCGTTC-3′); *ghrelin-2* (forward, 5′-GCTACTGACATAACAGCCACATC-3′; reverse, 5′-GAGGCTGCCTCAGTAGGTCTG-3′); *ghrelin-3* (forward, 5′-CAGTATGCCAGTCATGGGTC-3′; reverse, 5′-GTCACTGATGTGGCTGTTATGTC-3′); *ghrelin-4* (forward, 5′-CAGACACTCTCATTATGGTCAG-3′; reverse, 5′-CTTCCAATAATGCACTGACCC-3).

### Luciferase assays

The 300 ng/μl *pax4* mRNA and 250 ng/μl pGL3-*ghrelin-1-luc* or pGL3-*ghrelin-2-luc* luciferase reporter plasmids were co-injected into the embryos at the one-cell stage. pGL3-Control, pGL3-*ghrelin-1m-luc* (a mutated sequence of *ghrelin-1*) and pGL3-*ghrelin-2m-luc* (a mutated sequence of *ghrelin-2*) were the control groups. Luciferase activity was measured using a GloMax 20/20 Luminometer (Promega) using the Dual-Luciferase Reporter Assay System (Promega). Firefly luciferase activities were normalised by *Renilla* luciferase activities in embryos co-injected with the 5 ng/μl vectors of pRL-CMV.

### Quantification and statistical analysis

Cell Counting and Co-localisation Analysis plug-ins in ImageJ (version 1.50d) were used to quantify the cell numbers by different fluorescent labels and DAPI-stained nuclei, and to analyse colocalisation between cells. All experiments comparing treatment samples were performed using randomly assigned siblings. After at least two repeats, data were analysed for statistical significance using the comparison of means and a two-tailed Student's *t*-test with GraphPad (version Prism 9.0.0). Variance for all groups of data was presented as ±s.e.m. No data were excluded from analyses. Sample sizes were chosen according to the estimation of effect sizes. The exact sample size (*n*), *P*-value for each experimental group and statistical tests were indicated in the figures and figure legends.

## Supplementary Material

10.1242/develop.201306_sup1 Supplementary information
