# Supplementary material for: Pax4-Ghrelin mediates the conversion of pancreatic ε-cells to β-cells after extreme β-cell loss in zebrafish
Source: Development. 2023 Mar 27;150(6):dev201306. doi: 10.1242/dev.201306 (PMC10112926; doi:10.1242/dev.201306)
Supplement: Supplementary information [file develop-150-201306-s1.pdf]

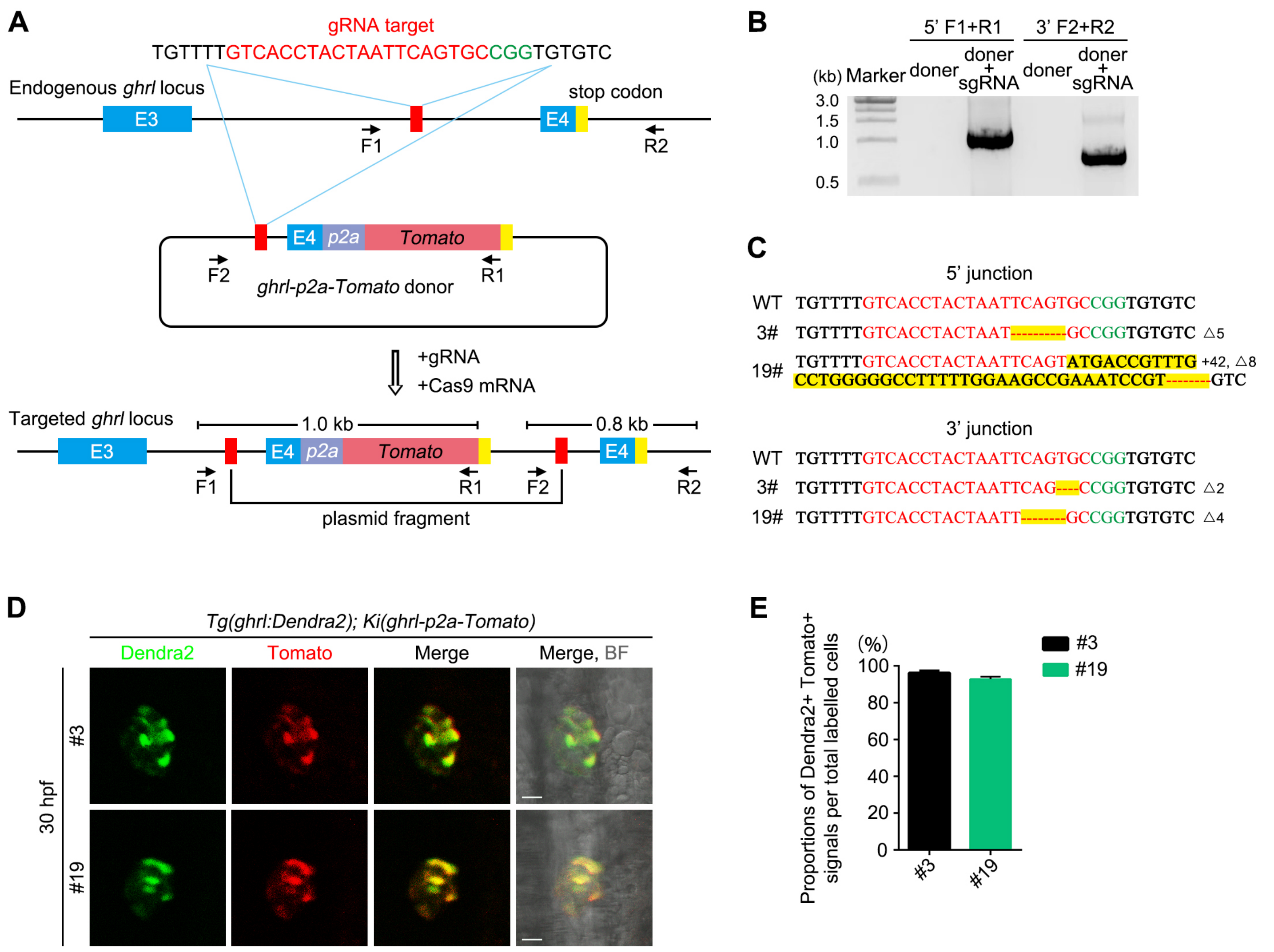

Fig. S1. Intron targeting-mediated knockin at endogenous *ghrelin* locus.

(A) The intron targeting-mediated strategy for generating Tomato knockin at the zebrafish *ghrelin* locus by the CRISPR/Cas9 system. The gRNA target sequence (red) and PAM site (green) were located at intron 3 between exon 3 (E3) and exon 4 (E4). The *ghrl-p2a-Tomato* cassette was integrated into the *ghrelin* locus after co-injection of the donor with the gRNA and Cas9 mRNA. (B) PCR analysis of the 5' and 3' junctions in the founder. The F1, R1, F2 and R2 primers are shown in (A). (C) 5' and 3' junction sequences of two *ghrl-p2a-Tomato* knockin founders #3 and #19. The indels are highlighted in yellow. (D) After crossing F1 offspring of *Ki(ghrl-p2a-Tomato)* founders with *Tg(ghrl:Dendra2)*, living images showed that Tomato signalling (red) was co-localised with ghrl:Dendra2 signals (green). (E) Statistical diagram of proportions of Dendra2+ Tomato+ signals per total labelled cells from offspring of different founders (n=20). Statistical data are expressed as mean±SEM, and p-values are calculated using Student's t-test. Scale bars, 10 μm.

Development • Supplementary information

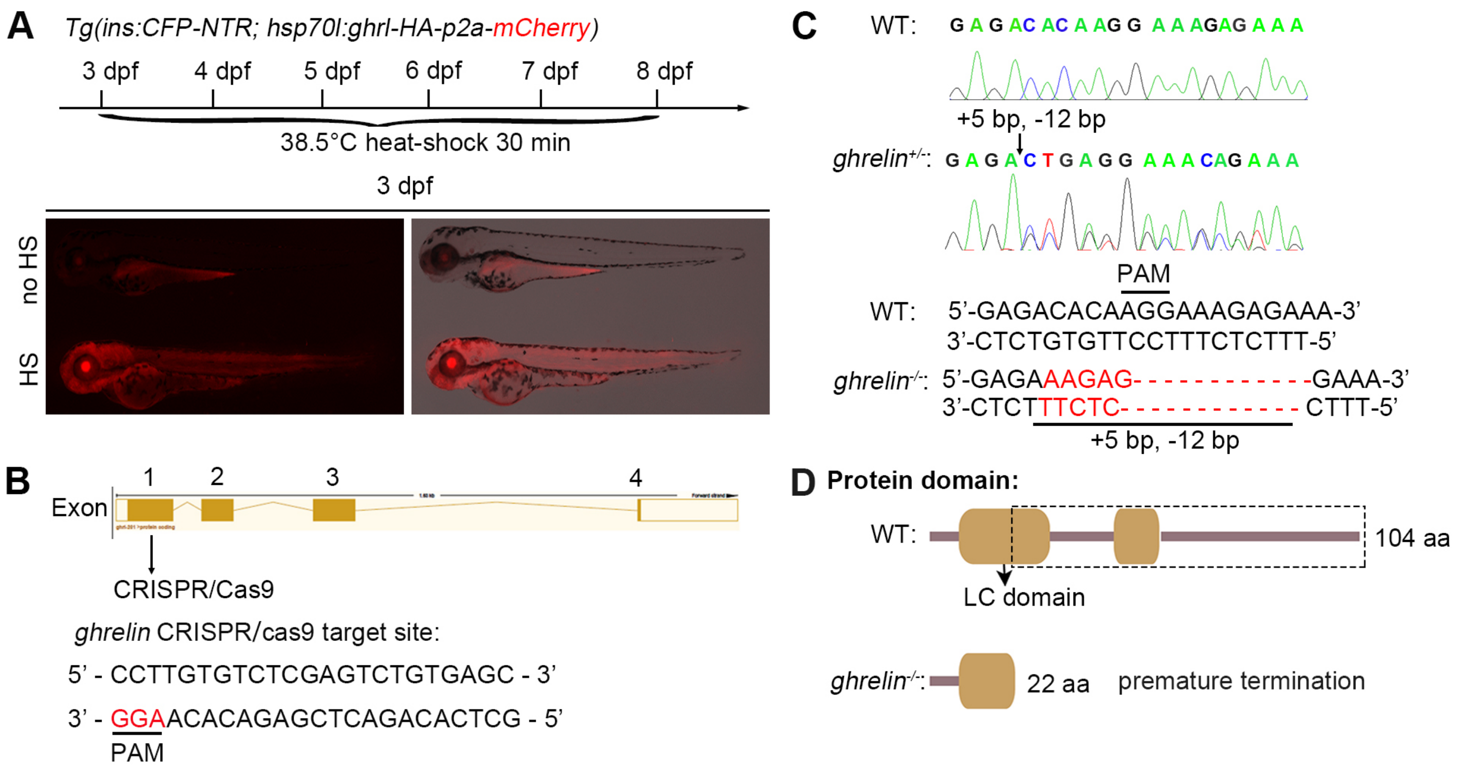

Fig. S2. Generation of *ghrelin* overexpression transgenic line and *ghrelin* CRISPR mutant.

(A) After heat-shock treatment per day from 3 dpf to 8 dpf, the *Tg(ins:CFP-NTR; hsp70l:ghrl-HA-p2a-mCherry)* double transgenic larvae displayed whole-mount expression of *ghrelin-HA-p2a-mCherry*. HS, heat-shock. (B) Schematic representation of the *ghrelin* gene. The CRISPR/Cas9 target site was located in exon 1 of *ghrelin*. (C and D) The 5-bp insertion and 12-bp deletion (C, +5 bp and -12 bp) in *ghrelin*$^{-/-}$ mutant led to formation of premature termination codon and truncation of the ghrelin low-complexity (LC) domain (D). The dotted box indicates the deleted protein domains.

Development • Supplementary information

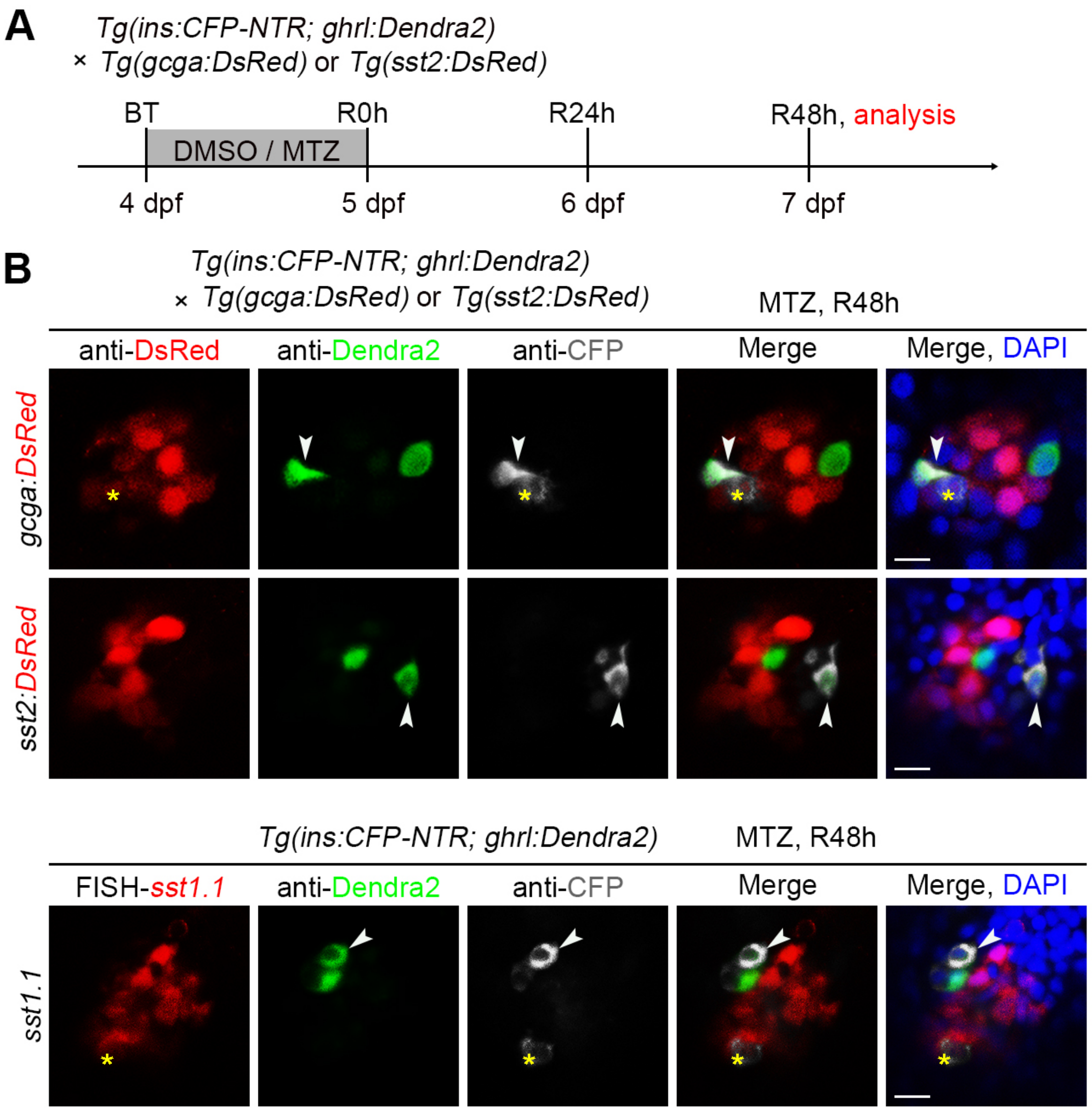

Fig. S3. Gcga, sst2 and *sst1.1* are not expressed in insulin and ghrelin double-positive cells during β-cell regeneration.

(A) Experimental scheme for MTZ treatment of the indicated transgenic lines. These larvae at 4–5 dpf were treated with MTZ for 24 hours and analysed at R48h after withdrawal of MTZ. (B) Analysis of regeneration showed except for sst2+ δ-cells, some gcga+ α-cells and sst1.1+ δ-cells could overlap with part of neogenic ins:CFP+ cells (asterisks), but not with the ghrl:Dendra2+ and ins:CFP+ double-positive cells (arrowheads). Scale bars, 10 µm.

Development • Supplementary information

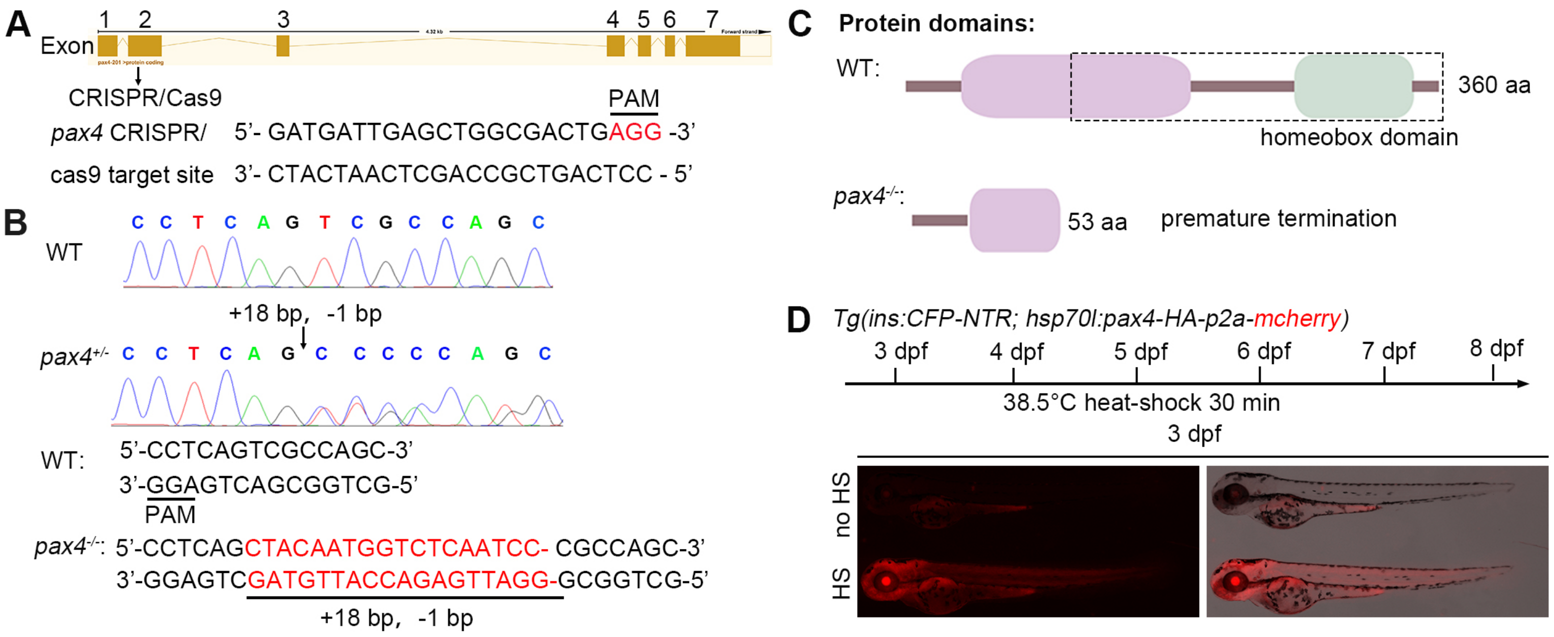

Fig. S4. Generation of *pax4* CRISPR mutant and *pax4* overexpression transgenic line. (A) Schematic representation of the *pax4* gene. The CRISPR/Cas9 target site was located in exon 2 of *pax4*. (B and C) The 18-bp insertion and 1-bp deletion (B, +18 bp and -1 bp) in *pax4*$^{-/-}$ mutant led to formation of premature termination codon and loss of the Pax4 homeobox domain (C). The dotted box indicates the deleted protein domains. (D) After heat-shock treatment per day from 3 dpf to 8 dpf, the *Tg(ins:CFP-NTR; hsp70l:pax4-HA-p2a-mCherry)* double transgenic larvae displayed whole-mount expression of *pax4-HA-p2a-mCherry*. HS, heat-shock.

Development • Supplementary information

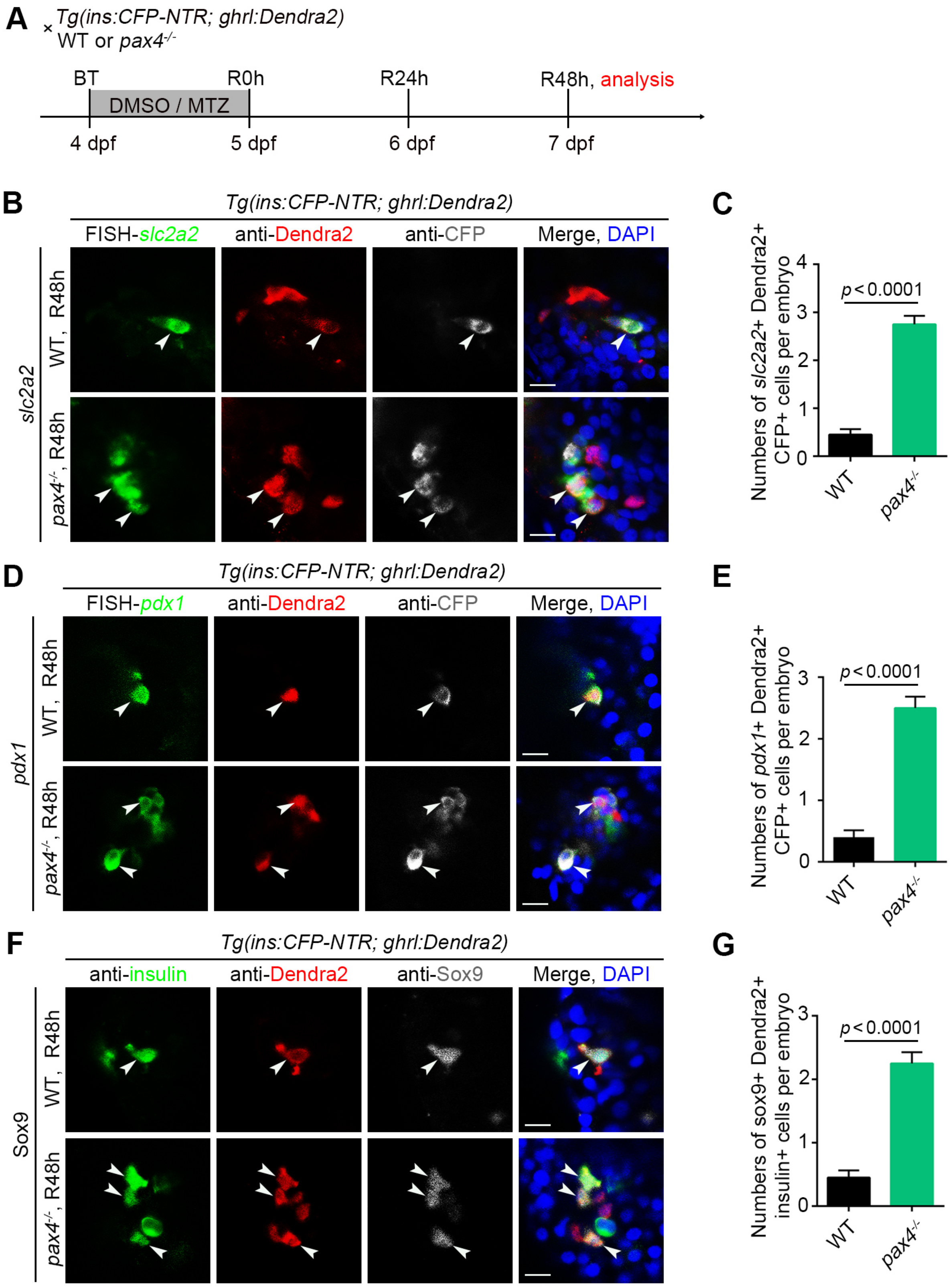

Development • Supplementary information

Fig. S5. The expression of *slc2a2*, *pdx1* and Sox9 in insulin and ghrelin double-positive cells after near-total β-cell ablation.

(A) Experimental scheme for MTZ treatment of the wild-type or *pax4*$^{-/-}$ mutant under *Tg(ins:CFP-NTR; ghrl:Dendra2)* double transgenic background. These larvae at 4–5 dpf were treated with MTZ for 24 hours and analysed at R48h after withdrawal of MTZ. (B-G) *slc2a2* (B), *pdx1* (D) and Sox9 (F) were transcribed in insulin/CFP+ and ghrl:Dendra2+ double-positive cells of the WT MTZ-treated groups, and their expressions were upregulated with the increase of neogenic β-cells in *pax4*$^{-/-}$ mutant. Arrowheads represent neogenic cells that are multi-signal co-localised. Statistical diagram of numbers of insulin/CFP+ Dendra2+ double-positive cells expressing *slc2a2* (C), *pdx1* (E) and Sox9 (G) per embryo at R48h (per group n=20). All statistical data are expressed as mean± SEM, and *p*-values are calculated using Student's t-test. Scale bars, 10 µm.

Development • Supplementary information

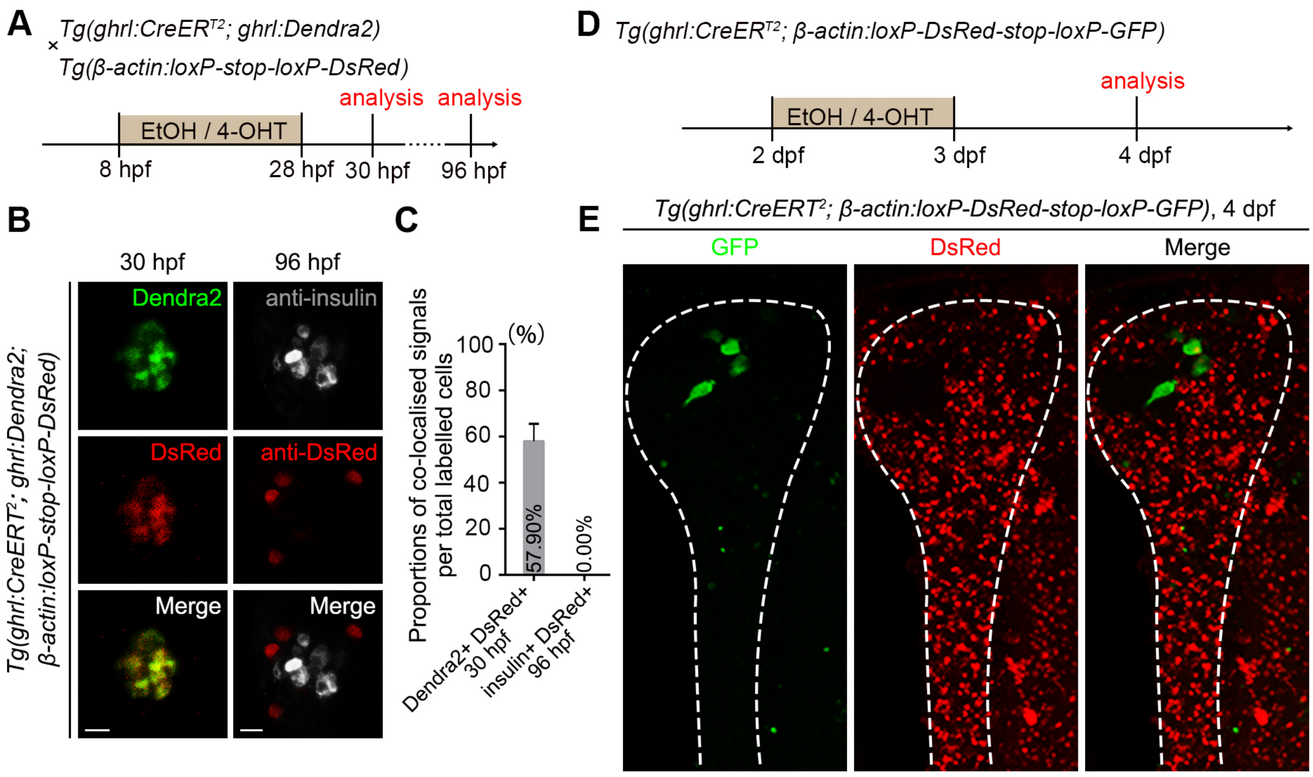

Fig. S6. The efficiency and specificity of ghrl:CreER^{T2} transgenic line.

(A) Experimental scheme for 4-OHT treatment of the *Tg(ghrl:CreER^{T2}; ghrl:Dendra2; β-actin:loxP-stop-loxP-DsRed)* triple transgenic lines. These larvae were treated with 4-OHT from 8 hpf and analysed at 30 hpf after drug withdrawal. (B) Living images showed that the majority of the DsRed+ signals overlapped with ghrl:Dendra2+ cells, but not insulin+ β-cells. (C) Statistical diagram of proportions of co-localised signals per total labelled cells (n=20). Statistical data are expressed as mean± SEM, and*p*-values are calculated using Student's t-test. (D) Experimental scheme for 4-OHT treatment of the *Tg(ghrl:CreER^{T2}; β-actin: loxP-DsRed-stop-loxP-GFP)* double transgenic lines. These larvae were treated with 4-OHT from 2 dpf and analysed at 4 dpf after drug withdrawal. (E) Living images showed GFP signals labelled the ghrelin-positive ε-cells, but not the other intrapancreatic cells. Scale bars, 10 µm.

Development • Supplementary information
